# A cassaine diterpene alkaloid, 3β-acetyl-nor-erythrophlamide, suppresses VEGF-induced angiogenesis and tumor growth via inhibiting eNOS activation

**DOI:** 10.18632/oncotarget.21307

**Published:** 2017-09-28

**Authors:** Nara Tae, Tran Manh Hung, Okwha Kim, Namho Kim, Suhyun Lee, Sunghun Na, Byung-Sun Min, Jeong-Hyung Lee

**Affiliations:** ^1^ Department of Biochemistry, College of Natural Sciences, Kangwon National University, Chuncheon, Republic of Korea; ^2^ College of Pharmacy, Catholic University of Daegu, Daegu, Republic of Korea; ^3^ Department of Obstetrics and Gynecology, Kangwon National University Hospital, School of Medicine, Kangwon National University, Chuncheon, Republic of Korea; ^4^ Current/Present address: Biomedical Science Department, VNUK Institute for Research & Executive Education, The University of Da Nang, Da Nang, Vietnam

**Keywords:** angiogenesis, cassaine diterpene alkaloid, VEGF, eNOS, HSP90

## Abstract

Angiogenesis is one of the hallmarks of cancer, playing an essential role in tumor growth, invasion, and metastasis. 3β-Acetyl-nor-erythrophlamide (3-ANE), a cassaine diterpene alkaloid compound from *Erythrophleum fordii*, exerts various pharmacological effects, including antitumor activity. However, the effects of 3-ANE on tumor angiogenesis and its potential molecular mechanism are still unknown. Here, we demonstrated that 3-ANE inhibited the vascular endothelial growth factor (VEGF)-mediated proliferation, migration, invasion, and capillary-like tube formation of human umbilical vascular endothelial cells (HUVECs), without inducing apoptosis. We also found that 3-ANE blocked angiogenesis *in vivo*, and suppressed tumor angiogenesis and human lung adenocarcinoma growth in the xenograft tumor model. Furthermore, we showed that 3-ANE blocked VEGF-mediated endothelial nitric oxide synthase (eNOS) phosphorylation, vascular permeability and NO production in HUVECs, via disrupting the VEGF-induced association of eNOS and heat-shock protein 90 (HSP90). Our studies therefore provide the first evidence that 3-ANE inhibits tumor angiogenesis by inhibiting the VEGF-mediated eNOS activation and NO production, and 3-ANE could be a potential candidate in angiogenesis-related disease therapy.

## INTRODUCTION

Angiogenesis is defined as the generation of new vascular growth sprouting from pre-existing vessels [1, 2]. Physiological angiogenesis is a vital mechanism during embryonic development and natural wound healing. In contrast, pathological angiogenesis is involved in the development of many diseases, including cancers, proliferative retinopathy and rheumatoid arthritis [1, 2]. Angiogenesis is well documented as a fundamental process in the transition of tumors from a dormant state to a malignant state, and is one of the hallmarks of cancer, playing an essential role in tumor growth, invasion, and metastasis [3]. New blood vessels infiltrate tumors, furnishing them with oxygen and nutrients, and provide a route for tumor metastasis [3]. Therefore, therapies based on blocking angiogenesis may be an effective strategy for inhibiting tumor growth and metastasis [4].

Numerous growth factors and cytokines are involved in the regulation of angiogenesis. Among all the known angiogenic molecules, vascular endothelial growth factor (VEGF) is the crucial regulator of angiogenesis [5, 6]. VEGF acts on endothelial cells (ECs) as both a chemotactic and a mitogenic factor, via endothelial cell-specific receptors: VEGFR1 (Flt-1), VEGFR2 (Flk-1/KDR) and VEGFR3 (Flt-4), among which VEGFR2 is considered to be the major transducer of VEGF-mediated effects on ECs, such as induction of proliferation, survival, migration, and permeability [6, 7]. In ECs, VEGFR2 signaling activates a number of downstream signaling mediators, including phosphoinositide-3 kinase (PI3K)/AKT, phospholipase Cγ (PLCγ), p38 mitogen-activated protein kinase (MAPK), and extracellular-signal-regulated kinase-1/2 (ERK-1/2), which act in coordination to initiate the angiogenic process [7]. The PI3K/AKT pathway is responsible for the production of nitric oxide (NO) through the activation of endothelial NO synthase (eNOS) and NO release from endothelial cells, and is an essential mediator of VEGF-stimulated angiogenesis [8, 9, 10]. Heat-shock protein 90 (HSP90) also plays an essential role in NO production through the regulation of eNOS activity in ECs. Exposure of ECs to diverse eNOS agonists such as VEGF, induces an increased association of HSP90 with eNOS and eNOS phosphorylation by Akt, leading to elevation of NO production [11, 12, 13]. Indeed, inhibition of either HSP90 or Akt results in a marked reduction of NO production [14].

3β-Acetyl-nor-erythrophlamide (3-ANE) is a group of the cassaine diterpene alkaloids from *Erythrophleum fordii (*Leguminosae), which is used in traditional Chinese medicine, mainly affecting invigoration and promoting blood circulation [15]. Cassaine diterpenoid amines and amides (customarily called cassaine alkaloids or *Erythrophleum* alkaloids) are the main constituents isolated from the genus *Erythrophleum* [15, 16]. The biological activities of cassaine alkaloids are not well elucidated. The alkaloids have displayed a digitalis-like action on the heart [17], and exhibited cytotoxic and apoptosis-inducing activity against some tumor cell lines [15, 18]. We found that a series of the cassaine diterpene alkaloids from *E. fordii* inhibits tube formation of human umbilical vascular endothelial cells (HUVECs) on Matrigel [19]. However, the detailed mechanisms by which 3-ANE inhibits tumor angiogenesis are still unknown.

In the present study, we demonstrated that 3-ANE significantly inhibits the VEGF-induced endothelial cell proliferation, cell cycle progression, migration and tube formation. Also, it suppresses the VEGF-induced neovascularization in mouse Matrigel implantation model, and tumor-associated angiogenesis in a xenograft tumor model. We also found that 3-ANE suppresses the VEGF-induced eNOS activation by blocking the VEGF-stimulated association of eNOS and HSP90. Taken together, our data suggest that 3-ANE may function as a novel and potent angiogenesis inhibitor that suppresses VEGF-induced eNOS activation.

## RESULTS

### 3-ANE inhibits VEGF-induced proliferation of HUVECs

**Figure 1:**
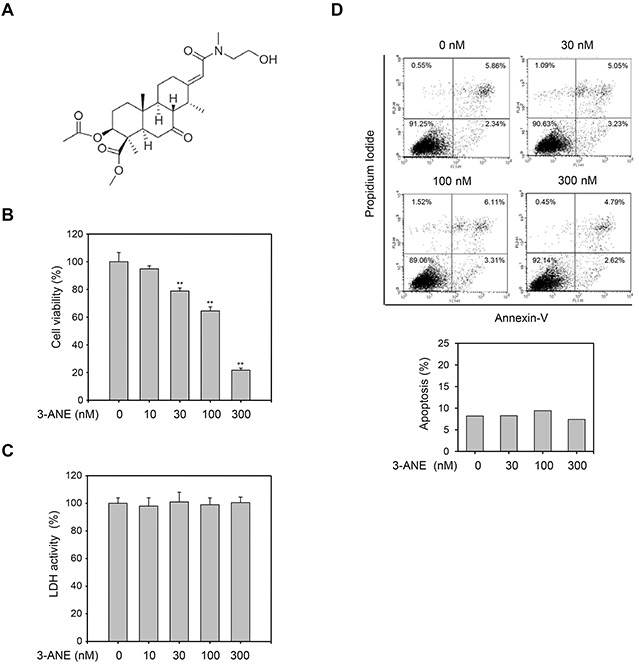
3-ANE inhibits proliferation of HUVECs without inducing apoptosis **(A)** Chemical structure of 3β-acetyl-nor-erythrophlamide (3-ANE). **(B)** HUVECs were incubated with the indicated concentrations of 3-ANE for 48 h and then cell proliferation was determined by MTT assay. *Columns*, mean of three independent experiments performed in triplicate; *bars*, S.D.; ^**^, *P*<0.01 *vs.* vehicle-treated control. **(C)** HUVECs were incubated with the indicated concentrations of 3-ANE for 48 h and then LDH activity in the culture medium was determined. *Columns*, mean of three independent experiments performed in triplicate. **(D)** HUVECs were incubated with the indicated concentrations of 3-ANE for 48 h, stained with Annexin-V/PI and then analyzed by a flow cytometry. The graphs represent the percentage of apoptosis performed in triplicate at the indicated concentrations (bottom).

To assess the antiangiogenic activity of 3-ANE, we first determined its anti-proliferative property. 3-ANE inhibited the proliferation of HUVECs in a concentration-dependent manner, with a half maximal inhibitory concentration of 105 ± 8 nM (Figure 1B). However, the medium LDH levels of 3-ANE-treated HUVECs did not change significantly (Figure 1C), suggesting that 3-ANE is not cytotoxic to HUVECs. Treatment of HUVECs with 3-ANE did not induce apoptosis or necrosis, up to 300 nM, as assessed by Annexin-V/PI double staining assay (Figure 1D). To further confirm that 3-ANE does not induce apoptosis in HUVECs, we performed another apoptosis assay by DAPI staining. DAPI staining assay also revealed that 3-ANE did not induce apoptosis in HUVECs (Supplementary Figure 1).

**Figure 2:**
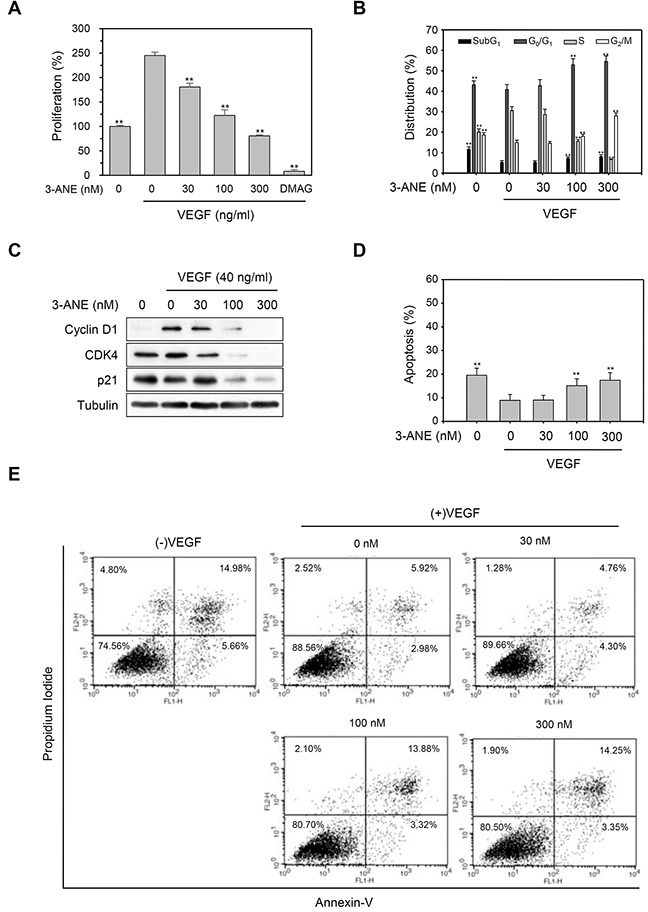
3-ANE inhibits VEGF-induced proliferation of HUVECs **(A)** HUVECs were incubated with the indicated concentrations of 3-ANE for 24 h with or without VEGF (40 ng/ml), and cell proliferation was determined by direct cell counting. DMAG (100 nM) was used as a positive control*. Columns*, mean of three independent experiments performed in triplicate; *bars*, S.D.; ^**^, *P*<0.01 *vs.* VEGF-only treated control. **(B)** HUVECs were incubated with the indicated concentrations of 3-ANE for 24 h with or without VEGF (40 ng/ml), and percent distribution of cells in each stage of the cell cycle was analyzed by flow cytometry with PI staining. *Columns*, mean of three independent experiments performed in triplicate; *bars*, S.D.; ^**^, *P*<0.01 *vs.* VEGF-only treated control. **(C)** HUVECs were incubated with the indicated concentrations of 3-ANE for 24 h with or without VEGF (40 ng/ml). Whole cell lysates were blotted with the indicated antibodies. **(D** and **E)** HUVECs were incubated with the indicated concentrations of 3-ANE for 24 h with or without VEGF (40 ng/ml), stained with Annexin-V/PI and then analyzed by a flow cytometry. The graphs represent the percentage of apoptosis performed in triplicate at the indicated concentrations (D); *bars*, S.D.; ^**^, *P*<0.01 *vs.* VEGF-only treated control. Representative flow cytometry histograms are shown (E).

Since VEGF plays a critical role in both physiological and pathological angiogenesis [5, 6], we next determined whether 3-ANE inhibits the VEGF-induced proliferation of HUVECs. 3-ANE significantly inhibited the VEGF-induced proliferation of HUVECs in a concentration-dependent manner, as determined by direct cell counting (Figure 2A) and MTT assay (Supplementary Figure 2). As a positive control, we used 17-dimethylaminoethylamino-17-demethoxygeldanamycin (DMAG), which is known to inhibit VEGF-induced proliferation and migration of HUVECs [20]. Cell cycle distribution analysis also revealed that 3-ANE inhibited the cell-cycle progression in VEGF-stimulated HUVECs (Figure 2B). Treatment of HUVECs with VEGF decreased the populations of G_0_/G_1_- and G_2_/M-phase, but increased the S-phase population. Treating with 3-ANE decreased the S-phase population and induced cell cycle arrest at the G_0_/G_1_- and G_2_/M-phase, in a concentration-dependent manner. Western blot analysis also revealed that 3-ANE inhibited the VEGF-induced accumulation of cyclin D1 in a concentration-dependent manner (Figure 2C). Interestingly, 3-ANE also decreased the expression levels of CDK4 and p21. To further determine whether the cytotoxic effect was attributable to exposure to 3-ANE in VEGF-treated HUVECs, we determined whether 3-ANE induces apoptosis in VEGF-treated HUVECs (Figure 2D and 2E). Treatment with VEGF suppressed the apoptosis of HUVECs induced by serum starvation; however, 3-ANE significantly inhibited the protective effect of VEGF on apoptosis induced by serum starvation in HUVECs. These results suggest that 3-ANE may inhibit VEGF-induced proliferation of HUVECs without inducing apoptosis.

### 3-ANE inhibits VEGF-induced migration, invasion, and tube formation of HUVECs and angiogenesis *in vivo*

**Figure 3:**
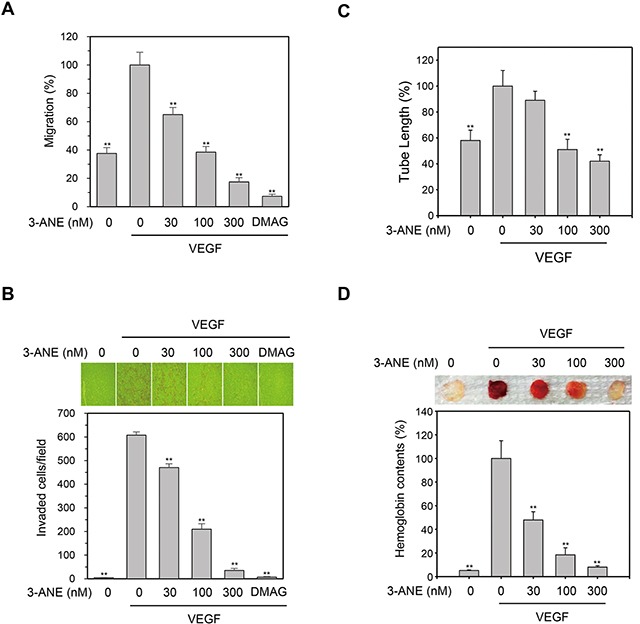
3-ANE inhibits VEGF-induced migration, invasion, tube formation of HUVECs and angiogenesis *in vivo* **(A)** HUVECs were treated with the indicated concentrations of 3-ANE and then underwent a wound-healing assay in the absence or presence of VEGF (40 ng/ml) for 24 h. DMAG (100 nM) was used as a positive control*. Columns*, mean of two independent experiments performed in triplicate; *bars*, S.D.; ^**^, *P*<0.01 *vs.* VEGF-only treated control. **(B)** HUVECs were treated with the indicated concentrations of 3-ANE and then underwent a Transwell invasion assay in the in the absence or presence of VEGF (40 ng/ml) for 24 h. DMAG (100 nM) was used as a positive control. Representative images are shown (top). The graphs represent quantification of invaded HUVECs (bottom). *Columns*, mean of two independent experiments performed in triplicate; *bars*, S.D.; ^**^, *P*<0.01 *vs.* VEGF-only treated control. **(C)** HUVECs were treated with the indicated concentrations of 3-ANE and a tube formation assay was performed in the presence or absence of VEGF (40 ng/ml) for 18 h. *Columns*, mean of three independent experiments performed in duplicate; *bars*, S.D.; ^**^, *P*<0.01 *vs.* VEGF-only treated control. **(D)** C57BL/6 mice were implanted subcutaneously with Matrigel plugs containing indicated concentrations of 3-ANE with or without VEGF. After 14 days, Matrigel plugs were excised. Representative photos are shown (top). Excised matrigel plugs were homogenized and the hemoglobin level was determined by Drabkin Reagent (bottom). *Columns*, mean of two independent experiments performed in triplicate; *bars*, S.D.; ^**^, *P*<0.01 *vs.* VEGF-only treated control.

Cell migration is a key step for endothelial cells in angiogenesis. We thus performed wound healing assays to evaluate whether 3-ANE affects the VEGF-induced migration of HUVECs. We observed a significant, concentration-dependent reduction in the number of migrated cells when HUVECs were treated with 3-ANE (Figure 3A). We also observed that 3-ANE inhibited the VEGF-induced invasion of HUVECs in a concentration-dependent manner (Figure 3B).

Angiogenesis is a complex process, and tube formation of endothelial cells is an essential step during angiogenesis. To further assess the antiangiogenic effects of 3-ANE, a two-dimensional Matrigel tube formation assay was performed. VEGF significantly increased the capillary-like network; however, treatment of 3-ANE decreased the formation of the network in a concentration-dependent manner (Figure 3C). We then evaluated the antiangiogenic effects of 3-ANE in an *in vivo* mouse Matrigel plug model of angiogenesis (Figure 3D). After being embedded subcutaneously into mice for 2 week, the Matrigel plugs containing VEGF alone appeared dark red, indicating that infiltrating vasculatures had formed inside the Matrigel via angiogenesis. In contrast, addition of 3-ANE to the Matrigel plugs concentration-dependently inhibited new vessel formation, with the Matrigel plugs being pale and weak in color. We next quantified the level of angiogenesis by determining the hemoglobin content of the plugs (Figure 3D). The 3-ANE-treated plugs showed a marked reduction in neovascularization, when compared with vehicle-treated groups. These data indicate that 3-ANE concentration-dependently and significantly suppresses the angiogenesis *in vitro* and *in vivo*.

### 3-ANE inhibits the VEGF-induced eNOS phosphorylation and NO production

**Figure 4:**
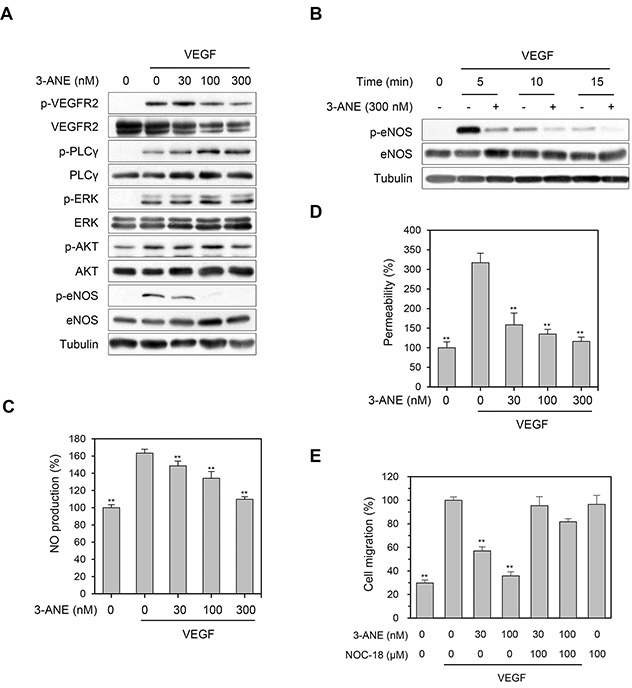
3-ANE inhibits VEGF-mediated eNOS phosphorylation and NO production **(A)** HUVECs were incubated with the indicated concentrations of 3-ANE for 10 min with or without VEGF (40 ng/ml). Whole cell lysates were blotted with the indicated antibodies. **(B)** HUVECs were incubated with 3-ANE (300 nM) for the indicated periods of time with or without VEGF (40 ng/ml). Whole cell lysates were blotted with the indicated antibodies. **(C)** HUVECs were incubated with the indicated concentrations of 3-ANE for 30 min with or without VEGF (40 ng/ml). NO production was determined by DAF2-DA. *Columns*, mean of two independent experiments performed in triplicate; *bars*, S.D.; ^**^, *P*<0.01 *vs.* VEGF-only treated control. **(D)** HUVECs were incubated with the indicated concentrations of 3-ANE for 60 min with or without VEGF (40 ng/ml) and then underwent a permeability assay with or without VEGF (40 ng/mL). *Columns*, mean of three independent experiments performed in triplicate; *bars*, S.D.; ^**^, *P*<0.01 *vs.* VEGF-only treated control. **(E)** HUVECs were treated with the indicated concentrations of 3-ANE or together with NOC-18 (100 μM) in the presence or absence of VEGF (40 ng/ml) and then cell migration assays were performed. *Columns*, mean of two independent experiments performed in triplicate; *bars*, S.D.; ^**^, *P*<0.01 *vs.* VEGF-only treated control.

Since VEGFR2 is considered a major mediator of several physiological and pathological effects of VEGF on endothelial cells [6, 7], we next determined whether 3-ANE inhibits the VEGF-induced phosphorylation of VEGFR2. 3-ANE did not significantly decrease the level of VEGF-induced VEGFR2 phosphorylation (Figure 4A). Moreover, 3-ANE did not inhibit VEGF-induced the phosphorylation of VEGFR2 downstream signaling molecules, such as PLC-γ, ERK, and AKT. Interestingly, we found that 3-ANE inhibited the VEGF-induced eNOS phosphorylation on Ser1177, which plays a critical role in eNOS activation [21], in a concentration- and time-dependent manner (Figure 4A and 4B). To further confirm the inhibitory effect of 3-ANE on VEGF-induced eNOS activation, we determined whether 3-ANE suppresses the VEGF-induced NO production and permeability of HUVEC monolayers. Intracellular NO levels were measured by loading cells with 10 μM of the NO-sensitive fluorescent probe DAF2-DA. VEGF stimulation of HUVECs increased the NO production; however, treating with 3-ANE significantly impaired the VEGF-induced NO production in a concentration-dependent manner (Figure 4C). Moreover, the ability of VEGF to increase the passage of FITC-labeled dextran across a monolayer of HUVECs was also blocked when HUVECs were treated with 3-ANE (Figure 4D). NO production in endothelial cells plays a predominant role in VEGF-induced angiogenesis [8, 22]. Thus, we next determined whether NOC-18, a NO donor, reverses the inhibitory effect of 3-ANE on VEGF-induced migration of HUVECs. Treatment with NOC-18 alone increased the migration of HUVECs; however, NOC-18 significantly abolished the inhibitory effect of 3-ANE on VEGF-induced migration of HUVECs (Figure 4E). Taken together, these results demonstrate that 3-ANE may inhibit the VEGF-induced angiogenesis via suppression of the VEGF-induced activation of eNOS and thereby NO production.

### 3-ANE impairs an association of eNOS with HSP90

**Figure 5:**
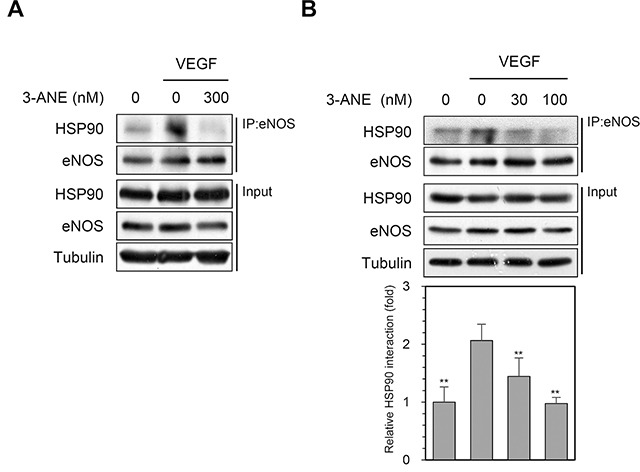
3-ANE inhibits an association of eNOS with HSP90 **(A** and **B)** HUVECs were incubated with the indicated concentrations of 3-ANE for 15 min with or without VEGF (40 ng/ml). Whole cell lysates were immunoprecipitated (IP) with eNOS antibody. The immunoprecipitates and input were blotted with the indicated antibodies. The graph represents quantification of HSP90 interaction with eNOS. *Columns*, mean of four independent experiments; *bars*, S.D.; ^**^, *P*<0.01 *vs.* VEGF-only treated control.

After stimulation of endothelial cells with agonists known to stimulate NO production, HSP90 associates with eNOS and plays an essential role in vascular NO production through the regulation of eNOS activity [11–13]. To further examine the inhibitory effect of 3-ANE on VEGF-induced NO production and eNOS phosphorylation, we determined whether 3-ANE regulates the association of eNOS with HSP90 in HUVECs, using the co-immunoprecipitation assays. VEGF stimulation markedly increased the association of eNOS with HSP90; however, treating with 3-ANE concentration-dependently impaired the VEGF-induced association of eNOS with HSP90 (Figure 5A and 5B). These results suggest that 3-ANE may inhibit the VEGF-induced eNOS activation by impairing the association of eNOS with HSP90.

### 3-ANE inhibits tumor growth and tumor angiogenesis in A549 xenograft mouse model

**Figure 6:**
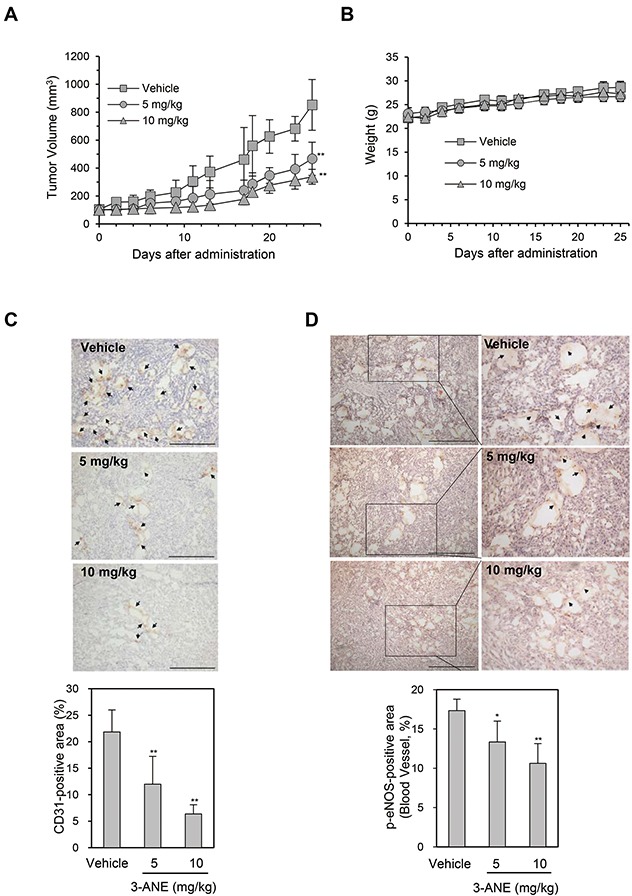
3-ANE inhibits tumor growth and tumor angiogenesis **(A)** A549 tumor-bearing mice were treated intraperitoneally with 3-ANE at 5 and 10 mg/kg or vehicle. Treatment with 3-ANE resulted in significant tumor growth inhibition *vs*. vehicle control. ^**^, *P*<0.01. **(B)** Body weight changes in 3-ANE and vehicle treated mice. **(C, D)** The tumor tissues were performed immunohistochemistry analysis using anti-CD31 (C) and anti-phospho-eNOS (D) antibody. Representative images of immunostaining results are shown. *Bar* = 100 μm. Arrows indicate positive staining. The graphs represent the quantification of CD31-positive area (n=10) and phospho-eNOS-positive area (n=10); *bars*, S.D.; ^**^, *P*<0.01; ^*^, *P*<0.05 *vs.* vehicle-treated control.

We next determined whether 3-ANE inhibits tumor growth and tumor angiogenesis using the A549 xenograft mouse model. 3-ANE significantly inhibited tumor growth in a dose-dependent manner. The average tumor size of the control group was 852 ± 182 mm^3^, whereas tumor size in 3-ANE-treated group at 10 and 5 mg/kg was 338 ± 53 and 466 ± 118 mm^3^, respectively (Figure 6A). However, no obvious differences were found in mouse body weight among the 3-ANE-treated groups and the control group (Figure 6B), suggesting that 3-ANE has low toxicity to the mouse at these dosage. We also evaluated the microvessel density in the A549 xenograft tissue sections by determining the number of CD31-positive blood vessels *in situ*. Analysis of CD31 immunostaining of the tissue sections showed a significant reduction of tumor microvessel density in 3-ANE-treated A549 tumors (Figure 6C). Moreover, the level of p-eNOS expression also significantly decreased in tumor microvessels of 3-ANE-treated A549 tumors (Figure 6D). These results suggest that 3-ANE may inhibit tumor growth by suppressing tumor angiogenesis in the A549 xenograft mouse model.

## DISCUSSION

In this study, we found that 3-ANE inhibited *in vivo* angiogenesis and suppressed key steps involved in angiogenesis, including proliferation, migration, invasion and capillary-like tube formation in endothelial cells. Further investigations revealed that 3-ANE suppressed the VEGF-induced eNOS activation and NO production by disrupting the association of eNOS and HSP90. In addition, we found that 3-ANE suppressed xenograft tumor growth and tumor angiogenesis. This is the first report showing that 3-ANE suppressed the VEGF-induced angiogenesis *in vitro* and *in vivo*, and tumor angiogenesis.

Angiogenesis is required for invasive tumor growth and metastasis, and antiangiogenic therapy is a novel therapeutic approach for cancer treatment [23, 24]. Among the numerous factors involved in angiogenesis, VEGF plays a crucial role in vessel sprouting and new vessel initiation through induction of proliferation, migration, vascular permeability, and survival of endothelial cells [25, 26]. The production of VEGF is increased in tumors because of oncogene activation, loss of tumor suppressor function, or changes in oxygen or glucose status. VEGFR2 is the major effector in ECs for execution of VEGF-stimulated cell proliferation, cell migration, and cell survival, leading to angiogenesis. Multiple pathways participate in the VEGF-induced angiogenesis [5, 7, 27]. The PI3K/AKT pathway activates the eNOS by phosphorylation and NO production in endothelial cells [28, 29]. Phosphorylation of eNOS by Akt leads to elevate NO production, which is a critical mediator for inducing angiogenesis [8–10]. An eNOS-specific inhibitor exhibits marked inhibition of pathological angiogenesis, vascular hyperpermeability and tumour growth [30]. Conversely, eNOS overexpression results in increased VEGF-dependent angiogenesis [31]. In the present study, we showed that 3-ANE significantly inhibited the VEGF-induced eNOS phosphorylation, NO production, and vascular permeability. Moreover, NOC-18, a NO donor, reversed the 3-ANE-mediated inhibition of HUVEC migration, suggesting that 3-ANE may exert its anti-angiogenic activity by inhibiting VEGF-induced eNOS activation.

A growing body of evidence shows that HSP90 serves as an essential chaperone protein to activate agonist-induced eNOS activation and NO production by mediating the AKT-eNOS interaction [11, 12, 13]. For example, HSP90 overexpression in endothelial cells is sufficient to enhance NO formation and activate eNOS by inducing its phosphorylation *in vitro* and *in vivo* [32, 33]. In contrast, inhibition of HSP90 activity effectively suppresses angiogenesis and tumor growth [14, 20, 34, 35]. We showed that 3-ANE suppressed the VEGF-induced eNOS phosphorylation and NO production in endothelial cells, and inhibited the association of eNOS and HSP90, indicating that 3-ANE inhibits the VEGF-induced angiogenesis by impairing the eNOS-HSP90 association, which is an essential role in eNOS phosphorylation by AKT and subsequent activation. These findings raise intriguing questions as to how 3-ANE regulates VEGF-induced association of eNOS with HSP90. We did not determine whether 3-ANE can directly bind to HSP90. However, we compared 3-ANE with geldanamycin, a well-known HSP90 inhibitor, for HSP90 client protein expression. Among HSP90 client proteins tested [36], 3-ANE did not significantly induce the degradation of RAF-1 and AKT unlike geldanamycin, but induced the degradation of CDK4 in HUVECs (Supplementary Figure 3). Thus, it is likely that 3-ANE may modulate the function of HSP90 differently from geldanamycin. However, the detailed mechanisms by which 3-ANE impairs VEGF-induced association of HSP90 and eNOS remain to be elucidated.

We demonstrated that 3-ANE significantly suppressed tumor growth via inhibiting tumor angiogenesis. Tumor angiogenesis is a complex multistep process. Inhibition of any step of these processes may lead to the disruption of angiogenesis. In our study, we demonstrated that 3-ANE suppressed HUVEC cell migration, capillary-like tube formation and proliferation, suggesting that 3-ANE affected the angiogenesis by directly targeting endothelial cells. In conclusion, we demonstrated for the first time that 3-ANE, a cassaine diterpene alkaloids, is a novel tumor angiogenesis inhibitor which targets the eNOS activation. We thereby conclude that 3-ANE may be a good candidate as an antiangiogenic agent, and a valuable lead compound for further development.

## MATERIALS AND METHODS

### Cells and cell culture

Human umbilical endothelial cells (HUVECs) were cultured on gelatin-coated plates in Medium 199 supplemented with penicillin-streptomycin (Invitrogen, Carlsbad, CA, USA), 20% heat-inactivated fetal bovine serum (FBS, Hyclone, Logan, UT, USA), heparin (Sigma-Aldrich, St Louis, MO, USA), and basic fibroblast growth factor (Millipore, Billerica, MA, USA). HUVECs were cultured in a humidified chamber at 37°C in a 5% CO_2_ atmosphere.

### Isolation of 3β-acetyl-nor-erythrophlamide

3β-Acetyl-nor-erythrophlamide (3-ANE) was isolated from *Erythrophleum fordii as* previously described [19] and its structure was shown in Figure 1A. 3-ANE was obtained as a white amorphous powder, and showed a [M]^+^ peak at *m/z* 477 by electron ionization-mass spectrometry. Its structure is shown in Figure 1A. The purity of 3-ANE was evaluated by ^1^H and ^13^C nuclear magnetic resonance spectra, which revealed very pure signals without any other impurities (Supplementary Figure 4 and Figure 5). 3-ANE was solubilized in 100% dimethyl sulfoxide, and used at a final concentration of less than 0.05% dimethyl sulfoxide.

### Antibodies and reagents

Anti-VEGFR2, anti-PLCγ, anti-HSP90, anti-Raf-1, anti-cyclin D1, anti-CDK4 and anti-p21 antibodies were purchased from Santa Cruz Biotechnology (Santa Cruz, CA, USA). Anti-VEGFR2, anti-phospho-VEGFR2 (Y1175), anti-phospho-PLCγ (Y783), anti-phospho-Erk (T202/Y204), anti-Erk, antiphospho-Akt (S473) and anti-Akt antibodies were purchased from Cell Signaling Technology (Danvers, MA, USA). Anti-eNOS and anti-phospho-eNOS (S1177) antibodies were purchased from BD Biosciences (San Diego, CA, USA). Anti-α-tubulin antibody was purchased from Sigma-Aldrich. Recombinant human VEGF165 and 4,5-diaminofluorescein diacetate (DAF2-DA) and DAPI (4′,6-diamidino-2-phenylindole dihydrochloride hydrate) were purchased from Sigma-Aldrich. DMAG (17-dimethylaminoethylamino-17-demethoxygeldanamycin) and NOC-18 was purchased from Santa Cruz Biotechnology.

### Cell proliferation and cytotoxicity assay

Cell proliferation was determined by direct cell counting with a hemocytometer and the MTT (3-(4,5-dimethylthiazol-2-yl)-2,5-diphenyl tetrazolium bromide) assay. In brief, HUVECs were seeded into 96-well plates (5 × 10^4^ cells/well), and then incubated with the indicated concentrations of 3-ANE for 48 h. To determine the effect of 3-ANE on VEGF-induced proliferation, HUVECs were seeded into 96-well plates (5 × 10^4^ cells/well) and serum-starved for 4 h. Serum-starved HUVECs were pretreated with various concentrations of 3-ANE for 30min in serum-free medium, and then stimulated with VEGF (40 ng/ml) for 24 h. For direct cell counting, cells were trypsinized and counted using a hemocytometer. For MTT assay, MTT (0.5 mg/ml) was added to each well for 3 h. The insoluble formazan products were then dissolved in DMSO, and the absorbance was determined at 540 nm. Cytotoxicity assay was performed by measuring lactate dehydrogenase (LDH) activity in cell culture supernatants. HUVECs were incubated with various concentrations of 3-ANE for 48 h, and then cell supernatants were recovered and determined LDH activity using LDH cytotoxicity assay kit in according to manufacturer's instructions (Cayman Chemicals, Ann Arbor, MI, USA).

### Immunoprecipitation and western blotting

Immunoprecipitation and Western blotting were performed as described previously [37, 38]. Briefly, cells were lysed in lysis buffer [50 mM Tris-HCl, pH 7.4, 150 mM NaCl, 1 mM EDTA, 5 mM sodium orthovanadate, 1% NP-40 and protease inhibitor cocktail (BD Biosciences, San Diego, CA, USA)], and centrifuged at 15,000 rpm for 30 min, at 4°C. For immunoprecipitation, equivalent amounts of cell lysates were incubated with the appropriate antibodies, followed by incubation with protein A/G agarose beads. Immunoprecipitates were extensively washed and resolved by sodium dodecyl sulfate-polyacrylamide gel electrophoresis (SDS-PAGE), transferred, and probed with the appropriate antibodies. The signal was detected using an enhanced chemiluminescent system (Thermo Scientific Pierce, Rockford, IL, USA).

### Wound healing migration and invasion assays

Wound healing migration and invasion assay were performed as described previously [37, 38]. For wound healing migration assays, HUVECs were seeded into 12-well plates and incubated to form a monolayer for wound healing assay. A yellow plastic pipette tip was used to make wounds across the cell monolayer. Wounded cells were incubated with the indicated concentrations of 3-ANE in the absence or presence of VEGF for 16 h at 37°C. Phase-contrast photographs were obtained using an inverted phase-contrast microscope. Invasion assays were performed using Matrigel-coated Transwells according to the manufacturer's instructions (BD Biosciences). HUVECs (5 × 10^4^ cells/well) were seeded into the upper chambers of the filters coated with Matrigel (BD Biosciences) with 200 μl of Medium 199 (0.5% FBS), and the upper chambers were placed into the lower chambers of 24-well culture dishes containing 800 μl of the same medium mentioned above, with or without VEGF (40 ng/ml). Cells on the inserts were incubated with the indicated concentrations of 3-ANE for 24 h at 37°C. Invaded cells were fixed, stained, and counted in five randomly selected microscopic fields (×100) per filter.

### Cell cycle distribution analysis

HUVECs were pretreated with the indicated concentrations of 3-ANE for 30 min, stimulated with VEGF for 24 h, washed twice with PBS, and then centrifuged. Briefly, the pellet was fixed in 80% (vol/vol) ethanol for 30 min at -20°C, washed once with PBS, and resuspended in cold propidium iodide (PI) solution (50 μg/ml) containing RNase A (0.1 mg/ml) in PBS (pH 7.4) for 30 min in the dark. Flow cytometric analyses were performed using FACS Calibur (Becton Dickinson, San Jose, CA, USA). Forward light scatter characteristics were used to exclude the cell debris from the analysis. CellQuest software was used to analyze the data (Becton-Dickinson).

### Apoptosis assays

Annexin-V/PI double staining and DAPI staining assays were used to detect apoptosis. For annexin-V/PI double staining, HUVECs were treated with 3-ANE for the indicated periods of time in the presence or absence of VEGF. The cells were then trypsinized, collected, rinsed with PBS, centrifuged, resuspended in 100 μl of a binding buffer, and incubated with 5 μl FITC-labeled annexin-V and 5 μl PI for 15 min in the dark at room temperature. The HUVECs were analyzed by FACS Calibur (Becton & Dickinson Co., USA). After the appropriate markings for the negative and positive populations were set, the percentage of Annexin V^−^/PI^−^ (living cells), Annexin V^+^/PI^−^ (early apoptotic cells), and Annexin V^+^/PI^+^ (late apoptotic cells) staining were determined. For DAPI staining, HUVECs were treated with or without 3-ANE for 48 h. The cells were washed with PBS, fixed in 4% (v/v) formaldehyde for 15 min, and then permeabilized with 0.1% (v/v) Triton X-100 for 15 min. A 200 μl DAPI solution (1 μg/ml) was added into each well for 30 min at 37°C in the dark and thereafter visualized using a fluorescence microscope (Nikon Inc., Tokyo, Japan).

### Permeability assay

HUVECs were grown up to confluence on gelatin-coated inserts (0.4 μm polycarbonate membrane) of Transwell inserts (Costar, Corning, NY, USA). Cells on the inserts were treated with the indicated concentrations of 3-ANE, and incubated with VEGF and FITC-dextran (Sigma) for the final 60 min. The amount of FITC-dextran that diffused through the endothelial monolayer into the lower chamber was measured using a microplate fluorometer (Biotek, Winooski, VT, USA).

### Tube formation assay

Growth factor-reduced Matrigel (130 μl) was added into wells of 48-well plates, and polymerized for 30 min at 37°C. HUVECs (5 × 10^4^ cells/well) were then seeded on polymerized Matrigel and treated with the indicated concentration of 3-ANE for 18 h, with or without VEGF. Tubular networks in each well were photographed, and the formation of capillary-like networks was evaluated by measuring the length of capillary-like network using ImageJ software (NIH).

### Matrigel plug assay

Matrigel plug assay was performed as described previously [39]. Animal experiments were approved by the Kangwon National University Animal Care and Ethics Committee. Briefly, 0.5 ml of growth factor-reduced Matrigel (BD Biosciences) was combined in solution with VEGF and the indicated concentrations of 3-ANE. The gel solution was injected subcutaneously as a single plug in 6-week-old C57BL/6 mice. After 14 days, the implants were recovered, and hemoglobin content was measured using Drabkin's reagent, according to the manufacturer's instruction (Sigma).

### NO fluorometric assay

HUVECs were plated on gelatin-coated coverslips, and loaded with 10 μM of DAF2-DA, at 37°C for 30 min. The medium containing the loading dye was removed, replaced with Medium 199, incubated for 30 min at 37°C to ensure complete de-esterification, and then treated with the indicated concentrations of 3-ANE. NO synthesis was induced by stimulation with VEGF for 30 min. Fluorescence intensity was measured using a microplate fluorometer (Biotek, Winooski, VT, USA).

### Xenograft tumor growth assay and immunohistochemistry

This investigation was approved by the Animal Research Committee of Kangwon National University. A549 cells (5 × 10^6^ cells) were implanted subcutaneously on the right sides of the dorsal area of 4-week-old male nude mice. After the tumor volume reached =100 mm^3^, the mice (5 mice per group) were injected intraperitoneally with 3-ANE (10 mg/kg and 5 mg/kg) dissolved in dimethyl sulfoxide:chremophore-EL:PBS (1:1:8 by volume), or control vehicle, every 2 or 3 days. The growth of the tumor xenograft was evaluated every other day, by determining the tumor volume using digital caliper. Tumor growth was measured by the following equation: volume = (length × width^2^)/0.52. After 25 days, mice were sacrificed, and tumors were removed and embedded in paraffin. Staining with anti-CD31 (Abcam, Cambridge, United Kingdom) or anti-phospho-eNOS (BD Biosciences) was performed on 5 μm sections. Images were taken with a microscope, and the number of blood vessels was analyzed using ImageJ software (NIH).

### Statistical analysis

Data are expressed as the mean ± standard error of mean (SEM). Statistical comparisons were made with one-way analysis of variance (ANOVA), and the difference between the experimental groups was further compared by the Fisher least significant difference test. *P* < 0.05 was considered to indicate significance.

## SUPPLEMENTARY MATERIALS FIGURES AND TABLES


