# Supplementary material for: A cassaine diterpene alkaloid, 3β-acetyl-nor-erythrophlamide, suppresses VEGF-induced angiogenesis and tumor growth via inhibiting eNOS activation
Source: Oncotarget. 2017 Sep 28;8(54):92346–58. doi: 10.18632/oncotarget.21307 (PMC5696186; doi:10.18632/oncotarget.21307)
Supplement: Supplementary file 1 [file oncotarget-08-92346-s001.pdf]

A cassaine diterpene alkaloid, 3β-acetyl-nor-erythrophlamide, suppresses VEGF-induced angiogenesis and tumor growth via inhibiting eNOS activation

SUPPLEMENTARY MATERIALS

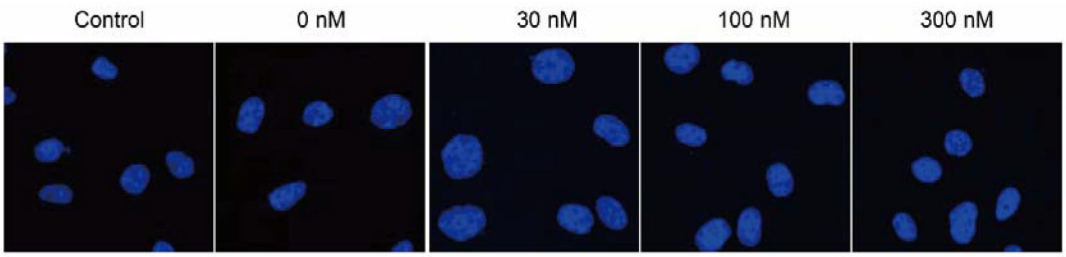

Supplementary Figure 1: Nuclear staining using DAPI of HUVECs in the presence of 3-ANE. HUVECs were incubated with the indicated concentrations of 3-ANE for 48 h, and then fixed and then stained with DAPI.

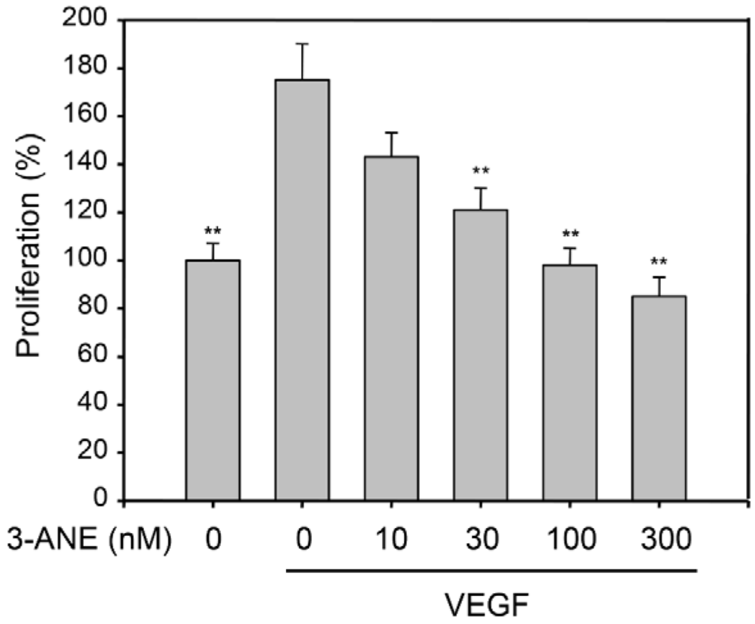

Supplementary Figure 2: 3-ANE inhibits VEGF-induced proliferation of HUVECs. HUVECs were incubated with the indicated concentrations of 3-ANE for 24 h with or without VEGF (40 ng/ml), and cell proliferation was determined by MTT assay.

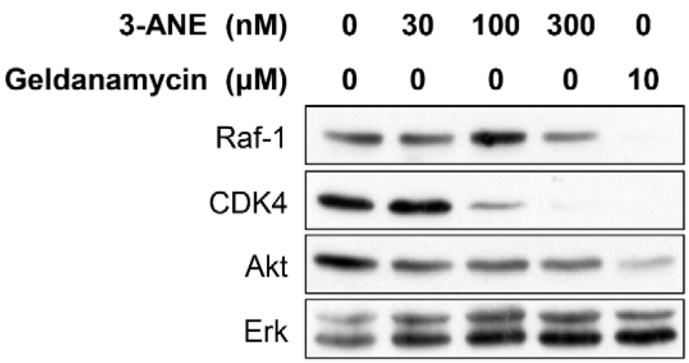

Supplementary Figure 3: Effect of 3-ANE and geldanamycin on the expression of some HSP90 client proteins in HUVECs. HUVECs were incubated with the indicated concentrations of 3-ANE for 24 h, and then whole cell lysates were blotted with the indicated antibodies.

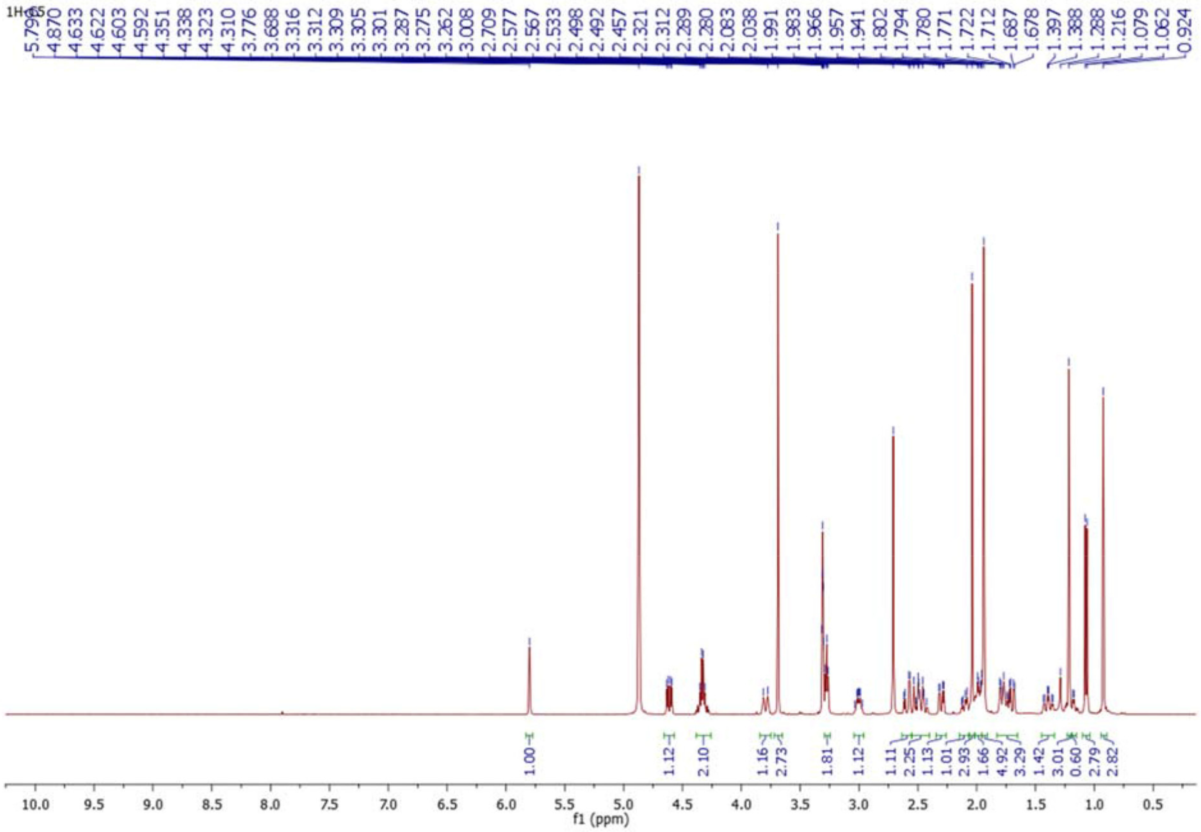

Supplementary Figure 4: ^{1}H NMR spectrum of 3-ANE in CD_3OD at 400 MHz.

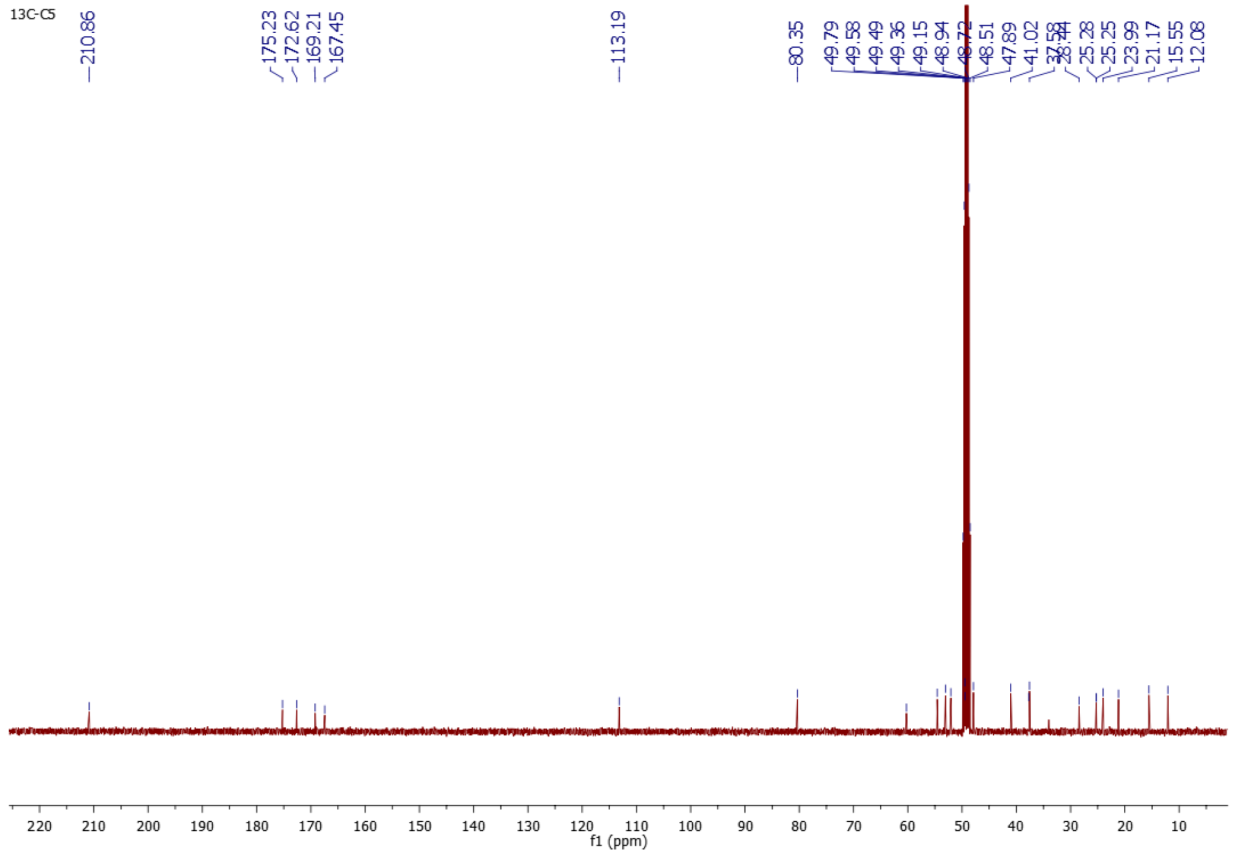

Supplementary Figure 5: ^{13}C NMR spectrum of 3-ANE in CD_3OD at 100 MHz.
